# Survival Status and Predictors of Mortality Among Preterm Neonates Admitted to the Neonatal Intensive Care Units in Central Ethiopia: A Prospective Follow‐Up Study

**DOI:** 10.1002/puh2.70123

**Published:** 2025-09-18

**Authors:** Daniel Tsega, Shegaw Geze Tenaw, Bogale Chekole, Abdulaziz Assefa, Mulugeta Animaw, Aberash Beyene Derribow, Mangistu Abera, Aynalem Belay

**Affiliations:** ^1^ Department of Midwifery College of Medicine and Health Science Wolkite University Wolkite Ethiopia; ^2^ Department of Nursing College of Medicine and Health Science Wolkite University Wolkite Ethiopia

**Keywords:** Ethiopia, preterm neonates, prospective follow‐up, survival status

## Abstract

**Introduction:**

Globally, 17.7% of under‐5 mortality and 36.1% of neonatal mortality occur due to preterm birth complications. Ethiopia is one of the top 10 countries with the highest neonatal mortality. Data on survival status and predictors of mortality among preterm neonates in Ethiopia remain limited. This study aimed to assess the survival status and predictors of mortality among preterm neonates admitted to the neonatal intensive care units of public hospitals in Central Ethiopia.

**Methods:**

A facility‐based prospective cohort study was conducted among 347 preterm neonates admitted to the neonatal intensive care units in selected public hospitals from October 1, 2022, to June 28, 2023. All admitted preterm neonates were enrolled. Data were collected using a structured questionnaire. The Kaplan–Meier curve was used to estimate the mean survival time and cumulative survival probability. To declare the associations, the Cox proportional hazards model was used to identify mortality predictors with adjusted hazard ratios with 95% confidence interval (CI) and *p* value.

**Results:**

Of 347 preterm newborns, 104 (29.97%) died, resulting in an incidence rate of 39.88 (95% CI: 32.90–48.33) fatalities per 1000 person‐day observations. Born from mothers with chorioamnionitis (AHR 3.89; 95% CI: 2.44, 6.18), born from mothers with gestational diabetes mellitus (GDM) (AHR 2.01; 95% CI: 1.27, 3.17), Apgar score at fifth minute less than 7 (AHR 1.87; 95% CI: 1.04, 3.36), having respiratory distress syndrome (RDS) (AHR 2.03; 95% CI: 1.14, 3.61), receiving kangaroo mother care (KMC) (AHR 1.86; 95% CI: 1.18, 2.94), and born less than 32 weeks of gestation (AHR 2.52; 95% CI: 1.27, 3.17) were significant predictors of mortality.

**Conclusions:**

Around one‐third of preterm neonates died. Improving the survival status should emphasize high‐risk neonates, with low fifth‐minute Apgar scores, having RDS, not receiving KMC, and neonates born to mothers with chorioamnionitis or GDM.

## Graphical abstract

Background:

-  17.7% of global under‐five deaths and 36.1% of neonatal deaths are due to preterm birth complications.
- Ethiopia ranks among the top 10 countries with the highest neonatal mortality.

To assess survival status and identify predictors of mortality among preterm neonates in NICUs of public hospitals in Central Ethiopia.

Study Design and Methods:

- Design: Facility‐based prospective cohort study
- Period: Oct 1, 2022–June 28, 2023
- Sample: 347 preterm neonates
- Analysis:
  - Kaplan–Meier curve for survival analysis
  - Cox proportional hazards model for identifying predictors (AHR, 95% CI)

Key Findings:

- Mortality rate: 29.97% (104 deaths)
- Incidence rate: 39.88 deaths per 1000 person‐days
- Significant predictors of mortality:
  - Chorioamnionitis (AHR 3.89)
  - Gestational diabetes mellitus (GDM) (AHR 2.01)
  - Apgar <7 at 5 min (AHR 1.87)
  - Respiratory distress syndrome (RDS) (AHR 2.03)
  - No Kangaroo Mother Care (KMC) (AHR 1.86)
  - Gestational age <32 weeks (AHR 2.52)

∼1 in 3 preterm neonates died. Focused interventions are needed for high‐risk groups, especially those with:

- Low Apgar scores
- RDS
- No KMC
- Born <32 weeks
- Maternal infections (chorioamnionitis)
- Maternal GDM.

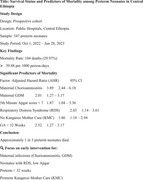

## 1 Introduction

Preterm birth is defined as the birth of a baby before 37 weeks of gestational age. Substantial progress in the reduction of neonatal mortality has been made globally in the last decades, although the decrease in neonatal mortality has remained unchanged in sub‐Saharan Africa [1]. The highest burden of newborn mortality occurs in middle‐ and low‐income countries [1]. Two‐thirds of neonatal mortality occurs within the first week of life, and the highest risk occurs within 24 h of birth. Four million perinatal and neonatal deaths occur every year [2]. The burden of stillbirth and early neonatal deaths is high in Ethiopia, making it one of the top 10 countries with the highest neonatal mortality [3, 4].

Globally, prematurity is the primary cause of death in children [5]. An estimated 15 million neonates, that is, more than 1 in 10 neonates, are born preterm annually [6]. Significant inequity exists in the burden of mortality among preterm infants around the globe. Globally, 17.7% of under‐5 mortality and 36.1% of neonatal mortality occur due to preterm birth complications [7]. The risk of neonatal mortality was 12 times higher in African countries [8]. In Ethiopia, 11% of under‐5 mortality occurs due to preterm birth [9]. Previous studies in Ethiopia revealed that preterm birth attributed 28.58%–76.0% of neonatal mortality [10, 11].

Different pieces of literature on neonatal mortality around the globe have revealed several predictors. Not receiving kangaroo mother care (KMC), having respiratory distress syndrome (RDS), not having ANC contact, neonatal sepsis, hypothermia, jaundice, timely initiation of breastfeeding, and maternal medical and obstetric complications are reported as the main predictors of neonatal mortality [10, 12, 13, 14, 15, 16, 17, 18, 19].

Despite efforts to scale up neonatal health, neonatal mortality remains high in Ethiopia, slightly increasing from 29 to 30 deaths per 1000 live births [9, 20]. Preterm birth contributes significantly to neonatal morbidity and mortality in Ethiopia [17, 21]. To achieve the sustainable development goals of neonatal health, which aim to reduce neonatal mortality to less than or equal to 12 deaths per 1000 live births by 2030, it is important to identify predictors of mortality tailored to preterm neonates [22].

Although several studies have been conducted on survival status and predictors of neonatal mortality [10, 12, 13, 14, 15, 16, 17], few have focused on preterm neonates [23, 24, 25]. In addition, existing literature on survival status and predictors of neonatal mortality among preterm infants relied on neonatal medical records [21, 23, 25, 26], which leads to missing some vital predictors and selection bias because those studies exclude charts with incomplete data. In addition, the possibility of medical record errors and diagnostic subjectivity is difficult to control in those studies. In this study, we did not rely on patient medical records; we did an observational prospective follow‐up study. The study aims to assess the survival status and predictors of mortality among preterm neonates admitted to the neonatal intensive care unit at Kembata and Halaba zones’ public hospitals.

## 2 Methods

### 2.1 Study Design, Setting, and Population

A longitudinal prospective cohort study was conducted in neonatal intensive care units of three public hospitals in the Kembata and Halaba zones from October 1, 2022, to June 28, 2023. There are five public hospitals in the Kembata zone and two public hospitals in the Halaba zone. The hospitals were selected on the basis of a high number of annual deliveries, having qualified consultants, and having established neonatal intensive care units. The study population consisted of neonates with a gestational age of less than 37 weeks at delivery and admitted to the NICU in selected hospitals in the Kembata and Halaba zones during the study period. The follow‐up was initiated during admission to the NICU until the study subjects either died, were discharged, left against medical advice, or were referred to another health facility. This study was an open cohort study, and any preterm neonates admitted to the NICU during the study period entered or left the study. During the entire period of the follow‐up, there were a total of 347 preterm neonates admitted to the NICU. We included all preterm neonates admitted to the NICU in the cohort.

### 2.2 Inclusion and Exclusion Criteria

We included neonates born before 37 weeks of gestation and admitted to the NICU during the study period. Neonates admitted to the NICU with an unknown gestational age during delivery were excluded from the study.

### 2.3 Data Measurements and Study Variables

The dependent variable was mortality (death).

Independent variables were as follows: sociodemographic predictors (maternal education status, maternal age, maternal occupation, residence, and marital status), maternal obstetrics, and medical predictors (number of parities, ANC follow‐up in index pregnancy, mode of delivery, place of birth, preeclampsia, APH, and gestational diabetes mellitus [GDM]), neonatal‐related predictors (gender, birth weight, Apgar score, gestational age at delivery, hypothermia, jaundice, hypoglycemia, RDS, seizure, sepsis, congenital anomalies, and birth trauma), and management‐related predictors (newborn resuscitation, KMC, oxygen, placed into incubator, antibiotics, anti‐seizure drugs, and phototherapy).

**Preterm birth**: Refers to a baby born alive before 37 completed weeks of pregnancy.
**Event**: The occurrence of death for preterm neonates from the first day of admission until the end of follow‐up.
**Censor**: A preterm baby other than dead (either discharged, left against medical advice, or referred to other health care).
**Survival time**: Refers to the time from admission to the NICU to the occurrence event or censoring.

### 2.4 Methods, Tools, and Procedures

A structured questionnaire was prepared by reviewing relevant literature [24, 25, 27, 28]. It includes maternal obstetrics and medical characteristics, neonatal complication‐related characteristics, neonatal treatment‐related predictors, the outcome of preterm neonates, and length of hospital stay. The first day of admission was taken as the starting time of follow‐up, whereas the last day of an event or censored occurrence was taken as the end of follow‐up time.

The study subjects were enrolled at admission and followed during their stay in the hospitals. During the follow‐up period, the data collectors recorded relevant clinical events until preterm neonates either became censored or died. Maternal‐related data were captured through interviews and reviewing medical records.

### 2.5 Data Quality Control

The principal investigator trained data collectors on the data collection process, study objectives, informed consent during the study period, and data confidentiality. A pre‐test was carried out on 5% of the population to ensure that the tool was appropriate. The lead investigator and supervisors double‐checked and examined the completed questionnaires to ensure that the collected data were complete and consistent.

### 2.6 Data Processing and Analysis

The collected data were coded and entered into EpiData version 3.1 and then exported to Stata version 14 for statistical analysis. There were no missing data for the variables included in the analysis. Frequency tables were used for categorical data, and mean with standard deviation (±SD) or median with interquartile range (IQR) for continuous variables. The Kaplan–Meier curve was used to estimate the mean survival time and cumulative survival probability. A log‐rank test was used to compare statistically significant differences across different covariates. The independent predictors of mortality were identified using survival analysis of the Cox proportional hazards model. The assumptions of the Cox regression were checked graphically and using the global test. Bivariate analysis, crude hazard ratio with 95% confidence interval (CI), was used to see the effect of each independent variable on the outcome variable. Variables that yielded a value ≤0.25 during bivariate analysis were entered into the multivariable analysis. The strength of association was measured using an adjusted hazard ratio with a 95% CI. A value of <0.05 was used as a cut point to declare significant predictors.

## 3 Results

### 3.1 Sociodemographic Characteristics

In this study, 294 (84.7%) mothers were married, and 213 (61.4%) mothers came from urban areas. Regarding the educational status of neonates’ mothers, 52 (15.0%) had no formal education, and 116 (33.4%) attended primary education. Regarding mothers’ occupations, 181 (52.2%) were housewives, and 84 (24.2%) were employed. Of 347 neonates, 179 (51.6%) neonates were female, and 49 (14.1%) neonates delivered at less than 32 weeks of gestation (Table 1).

**TABLE 1:** Sociodemographic characteristics of preterm neonates and their respective among preterm neonates admitted to the neonatal intensive care units of Kembata and Halaba Zone public hospitals, Central Ethiopia, 2023 (*n* = 347).

Variable	Category	Frequency (%)	Status	
			Death	Censored
			N (%)	N (%)
Marital status	Single	9 (2.59)	2 (22.2)	7 (77.9)
	Married	294 (84.7)	88 (29.9)	206 (70.1)
	Divorced	32 (9.2)	8 (25.0)	24 (75.0)
	Windowed	12 (3.46)	6 (50.0)	6 (50.0)
Mother’ educational status	No formal education	52 (15.0)	20 (38.4)	32 (61.5)
	Primary education	116 (33.4)	30 (25.9)	86 (74.1)
	Secondary education	98 (28.2)	26 (26.5)	72 (73.5)
	College and above	81 (23.4)	16 (19.8)	65 (80.3)
Mother’ occupation	Housewife	181 (52.2)	71 (39.2)	110 (60.8)
	Employed	84 (24.2)	15 (17.9)	69 (82.1)
	Merchant	82 (23.6)	18 (21.9)	64 (78.1)
Residence	Rural	134 (38.6)	58 (43.3)	76 (56.7)
	Urban	213 (61.4)	46 (21.6)	167 (78.4)
Sex of neonates	Male	168 (48.4)	57 (33.9)	111 (66.1)
	Female	179 (51.6)	47 (26.3)	132 (73.7)
Gestational age at delivery	<32	49 (14.1)	28 (57.1)	21 (42.9)
	32–34.6/7	188 (54.2)	61 (32.5)	127 (67.5)
	35–36.6/7	110 (31.7)	15 (13.64)	95 (86.4)

### 3.2 Obstetrics and Medical Characteristics

A great majority, 320 (92.2%) of the neonates’ mothers were multipara, and only 3 (0.9%) mothers were grand multipara. Of the total admitted preterm neonates, 80 (23.1%) of neonates’ mothers had a history of preterm delivery, 78 (22.5%) of neonates’ mothers had a history of abortion, and 302 (87.0%) of neonates’ mothers had planned pregnancies. Twenty (5.8%) mothers delivered at home, and 283 (81.6%) gave birth through SVD. Concerning obstetric complications in the index pregnancy, 75 (21.6%) had preeclampsia, 85 (24.5%) had PROM, 88 (25.4%) had chorioamnionitis, and 31 (8.9%) had APH. Regarding maternal medical disorders, 70 (20.2%) had GDM, and 51 (14.7%) were anemic (Table 2).

**TABLE 2:** Obstetrics and medical characteristics of preterm neonates and their respective mothers among preterm neonates admitted to the neonatal intensive care units of Kembata and Halaba Zone public hospitals, Central Ethiopia, 2023 (*n* = 347).

Variable	Category	Frequency (%)	Status	
			Death	Censored
			N (%)	N (%)
Number of parity	One	24 (6.9)	6 (25.0)	18 (75.0)
	Two‐four	320 (92.2)	97 (30.3)	223 (69.7)
	Five and above	3 (0.9)	1 (33.3)	2 (66.7)
History of preterm delivery	Yes	80 (23.1)	37 (46.3)	43 (53.7)
	No	267 (76.9)	67 (25.1)	200 (7.9)
History of abortion	Yes	78 (22.5)	41 (52.6)	37 (47.4)
	No	269 (77.5)	63 (23.4)	206 (76.6)
The desire for a recent pregnancy	Planned	302 (87.0)	86 (28.5)	216 (71.5)
	Unplanned	45 (13.0)	18 (40.0)	27 (60.0)
ANC in the recent pregnancy	Yes	324 (93.4)	93 (28.7)	231 (71.3)
	No	23 (6.6)	11 (47.8)	12 (51.2)
Pregnancy type	Single	328 (94.5)	91 (27.7)	237 (72.3)
	Multiple	19 (5.5)	13 (68.4)	6 (31.6)
Mode of delivery	SVD	283 (81.6)	65 (23.0)	218 (77.0)
	Instrumental	51 (14.7)	28 (54.9)	23 (45.1)
	Cesarean section	13 (3.7)	2 (15.4)	11 (84.6)
Place delivery	Home	20 (5.8)	10 (50.0)	10 (50.0)
	Health center	36 (10.4)	5 (13.9)	31 (86.1)
	Hospital	291 (83.8)	89 (30.6)	202 (69.4)
Preeclampsia	Yes	75 (21.6)	27 (36.0)	48 (64.0)
	No	272 (78.4)	77 (28.3)	195 (71.7)
PROM	Yes	85 (24.5)	59 (69.4)	26 (30.6)
	No	262 (75.5)	45 (17.2)	217 (82.8)
APH	Yes	31 (8.9)	10 (32.3)	21 (67.7)
	No	316 (91.1)	94 (29.8)	222 (70.2)
Chorioamnionitis	Yes	88 (25.4)	65 (73.9)	23 (26.1)
	No	259 (74.6)	39 (15.1)	220 (84.9)
GDM	Yes	70 (20.2)	47 (67.1)	23 (32.9)
	No	277 (79.8)	57 (20.6)	220 (79.4)
Anemia	Yes	51 (14.7)	21 (41.2)	30 (58.8)
	No	296 (85.3)	83 (28.0)	213 (72.0)

Abbreviation: GDM, gestational diabetes mellitus.

### 3.3 Neonatal Complications‐Related Characteristics

Among 347 preterm neonates, 231 (66.6%) were hypothermic, 101 (29.1%) had jaundice, 34 (9.8%) had an Apgar score at the fifth minute less than 7, and 220 (63.4%) developed sepsis. About 290 (83.6%) neonates were hypoglycemic, 105 (30.3%) developed RDS, and 42 (12.1%) neonates had birth trauma (Table 3).

**TABLE 3:** Neonatal complication‐related predictors among preterm neonates admitted to the neonatal intensive care units of Kembata and Halaba Zone public hospitals, Central Ethiopia, 2023 (*n* = 347).

Variable	Category	Frequency (%)	Status	
			Death	Censored
			N (%)	N (%)
Hypothermia	Yes	231 (66.6)	71 (30.7)	160 (69.3)
	No	116 (33.4)	33 (28.5)	83 (71.5)
Jaundice at admission	Yes	101 (29.1)	31 (31.7)	69 (68.3)
	No	246 (70.9)	72 (29.3)	174 (70.7)
Apgar scored in the fifth minute, less than 7	Yes	34 (9.8)	18 (52.9)	16 (47.1)
	No	313 (90.2)	86 (27.5)	227 (72.5)
Hypoglycemia	Yes	290 (83.6)	94 (32.4)	196 (67.6)
	No	57 (16.4)	10 (17.5)	47 (82.5)
Seizure	Yes	16 (4.6)	3 (18.7)	13 (81.3)
	No	331 (95.4)	101 (30.5)	230 (69.5)
Sepsis	Yes	220 (63.4)	67 (30.5)	153 (69.5)
	No	127 (36.6)	37 (29.1)	90 (70.90)
Birth trauma	Yes	42 (12.1)	21 (50.0)	21 (50.0)
	No	305 (87.9)	83 (27.2)	222 (72.8)
Congenital anomalies	Yes	5 (1.4)	1 (20.00)	4 (80.0)
	No	342 (98.6)	103 (30.1)	239 (69.9)
RDS	Yes	105 (30.3)	55 (52.4)	50 (47.6)
	No	242 (69.7)	49 (20.2)	193 (79.8)
	Yes	290 (83.6)	94 (32.4)	196 (67.6)
	No	57 (16.4)	10 (17.5)	47 (82.5)

Abbreviation: RDS, respiratory distress syndrome.

### 3.4 Treatment‐Related Predictors

About 331 (95.4%) neonates were treated with antibiotics, 100 (28.8%) neonates were under phototherapy, and 269 (77.5%) neonates received oxygen therapy (Table 4).

**TABLE 4:** Treatment‐related predictors among preterm neonates admitted to the neonatal intensive care units of Kembata and Halaba Zone public hospitals, Central Ethiopia, 2023 (*n* = 347).

Variable	Category	Frequency (%)	Status	
			Death	Censored
			N (%)	N (%)
Newborn resuscitation	Yes	110 (31.7)	56 (50.9)	54 (49.1)
	No	237 (68.3)	48 (20.3)	189 (79.7)
KMC	Yes	159 (45.8)	34 (21.4)	125 (78.6)
	No	188 (54.2)	70 (37.2)	118 (62.8)
Antibiotics	Yes	331 (95.4)	101 930.5)	230 (69.5)
	No	16 (4.6)	3 (18.8)	13 (81.20)
Phototherapy	Yes	100 (28.8)	31 (31.0)	69 (69.0)
	No	247 (71.2)	73 (29.5)	174 (70.5)
Blood transfusion	Yes	29 (8.4)	15 (51.7)	14 (42.3)
	No	318 (91.6)	89 (28.0)	229 (72.0)
Glucose	Yes	302 (87.0)	7 (15.6)	38 (84.40)
	No	45 (13.0)	97 (32.1)	67.9
Received oxygen	Yes	269 (77.5)	99 (36.8)	170 (63.2)
	No	78 (22.5)	5 (6.4)	73 (93.6)
The newborn entered the incubator	Yes	171 (49.3)	57 (33.3)	144 (66.70)
	No	176 (50.7)	47 (26.7)	129 (73.3)
The newborn took a seizure drug	Yes	23 (6.6)	3 (13.00)	20 (87.0)
	No	324 (93.4)	101 (31.2)	223 (68.8)
Oxygen	Yes	269 (77.5)	99 (36.8)	170 (63.2)
	No	78 (22.5)	5 (6.4)	73 (93.6)

Abbreviation: KMC, kangaroo mother care.

### 3.5 Survival and Mortality of Preterm Neonates

**FIGURE 1:**
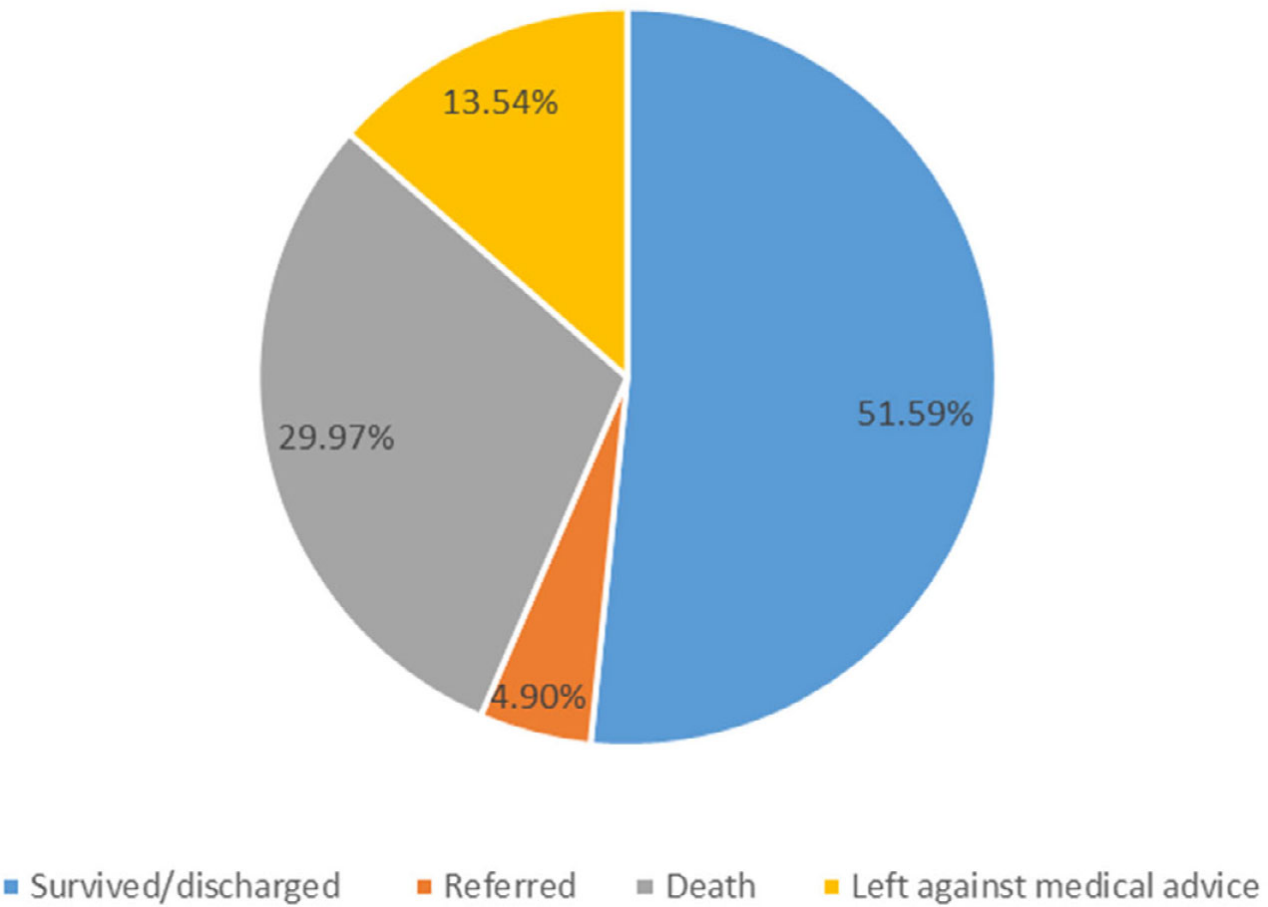
Outcomes of follow‐up among preterm neonates admitted to the neonatal intensive care units of Kembata and Halaba Zone public hospitals, Central Ethiopia, 2023 (*n* = 347).

A total of 396 preterm neonates were followed for up to 64 days (2608 person‐days) of age from the first day of admission to an event or censored occurrence. Of 347 preterm neonates in the cohort, 104 (29.97%) died with an incidence rate of 39.88 (95% CI: 32.90–48.33) deaths per 1000 person‐day observations. The highest incidence rate of mortality, 63.4 per 1000 person‐days, occurred on the first day of admission. Among 204 censored neonates, 51.59% were discharged with improvement, 13.54% were left against medical advice, and 4.90% were referred to another health facility (Figure 1).

**FIGURE 2:**
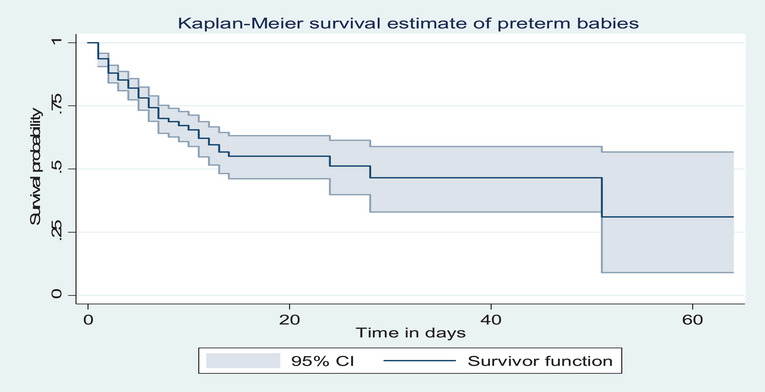
Overall Kaplan–Meier survival estimate among preterm neonates admitted to the neonatal intensive care units of Kembata and Halaba Zone public hospitals, Central Ethiopia, 2023 (*n* = 347).

The median survival time for preterm newborns was 28 days, with a range of 1–64 days. The cumulative survival rate was 93.6% on the first day of admission. The cumulative survival probabilities were 84.9% on the third day, 68.6% on the seventh day, and 29.7% at the end of the follow‐up period (Figure 2).

### 3.6 Survival Function and Comparison of Survival Probability for Different Categorical Variables

**FIGURE 3:**
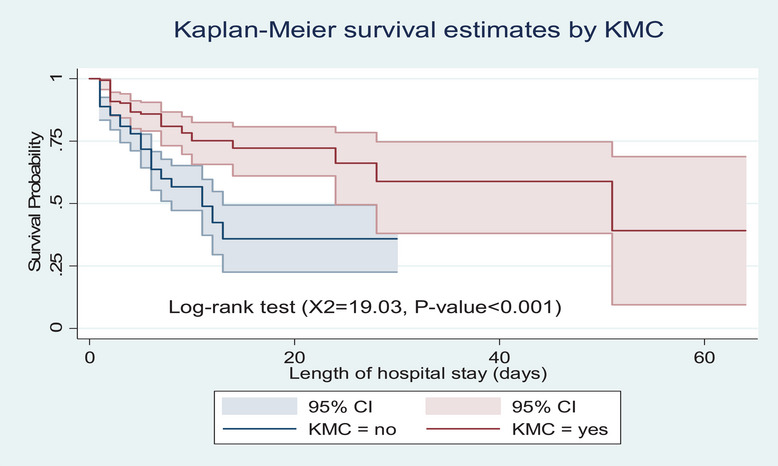
Kaplan–Meier survival estimate by KMC among preterm neonates admitted to the neonatal intensive care units of Kembata and Halaba Zone public hospitals, Central Ethiopia, 2023 (*n* = 347). CI, confidence interval; KMC, kangaroo mother care.

Preterm neonates who received KMC had a higher survival probability than those neonates who did not receive KMC (*p* value <0.001) (Figure 3).

**FIGURE 4:**
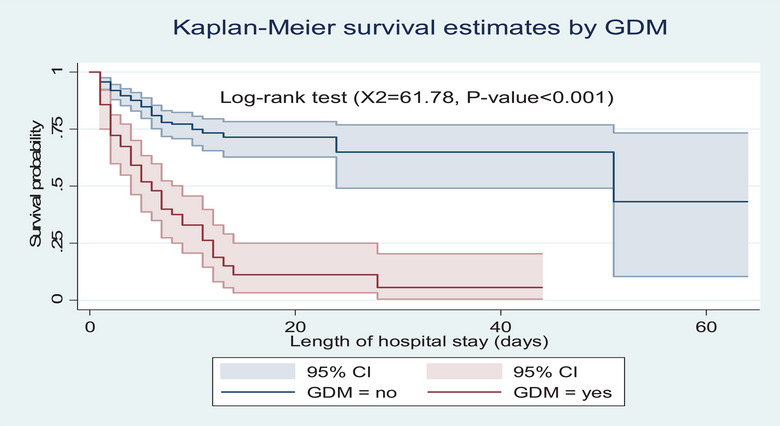
Kaplan–Meier survival estimate by maternal GDM among preterm neonates admitted to the neonatal intensive care units of Kembata and Halaba Zone public hospitals, Central Ethiopia, 2023 (*n* = 347). CI, confidence interval; GDM, gestational diabetes mellitus.

**FIGURE 5:**
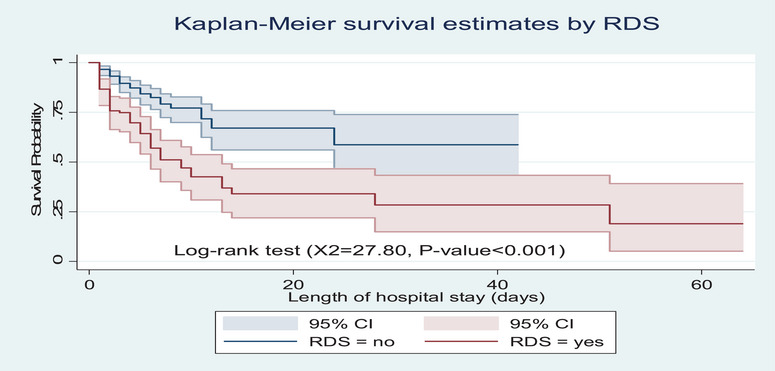
Kaplan–Meier survival estimate by RDS among preterm neonates admitted to the neonatal intensive care units of Kembata and Halaba Zone public hospitals, Central Ethiopia, 2023 (*n* = 347). CI, confidence interval; RDS, respiratory distress syndrome.

**FIGURE 6:**
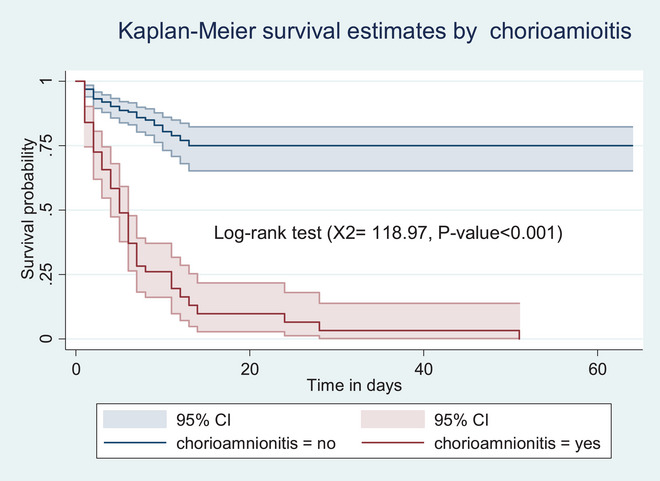
Kaplan–Meier survival estimate by chorioamnionitis among preterm neonates admitted to the NICU of Kembata and Halaba zones’ public hospitals, Central Ethiopia. CI, confidence interval.

However, preterm neonates born to mothers with GDM had a lower survival probability than their counterparts (*p* value <0.001) (Figure 4). Similarly, neonates with RDS had shorter survival experiences than their counterparts (*p* value <0.001) (Figure 5). In addition, preterm neonates born to mothers with chorioamnionitis had a lower survival probability than their counterparts (*p* value <0.001) (Figure 6).

### 3.7 Predictors of Mortality in Preterm Neonates

In multivariable Cox regression: Born from mothers with chorioamnionitis (AHR 3.89; 95% CI: 2.44, 6.18), born from mothers with GDM (AHR 2.01; 95% CI: 1.27, 3.17), Apgar score at fifth minute less than 7 (AHR 1.87; 95% CI: 1.04, 3.36), having RDS (AHR 2.03; 95% CI: 1.14, 3.61), received KMC (AHR 1.86; 95% CI: 1.18, 2.94), gestational age less than 32 weeks at delivery (AHR 2.52; 95% CI: 1.27, 3.17) were predictors of mortality in preterm neonates.

Neonates born to mothers with chorioamnionitis had 3.89 times more risk of death than those without chorioamnionitis. Neonates born to mothers with GDM also had 2.01 times more risk of mortality than their counterparts. Preterm neonates who did not receive KMC had 1.86 times shorter time to death than those who received KMC. Neonates with a fifth‐minute Apgar score of less than 7 had 1.87 times more risk of death. Similarly, neonates with RDS had a 2.03 times shorter time to death than neonates without RDS. Furthermore, neonates born less than 32 weeks of gestation had 2.52 times more hazards than neonates born between 35 and 36.6/7 weeks of gestation (Table 5).

**TABLE 5:** Predictors of mortality among preterm neonates admitted to the neonatal intensive care units of Kembata and Halaba Zone public hospitals, Central Ethiopia, 2023 (*n* = 347).

Variable	Category	Death	Censored	CHR (95% CI)	AHR (95% CI)	p > |z|
Mother’ educational status	No formal education	20	32	3.50 (1.92, 9.40)	1.42 (0.62, 3.27)	0.412
	Primary education	30	86	1.48 (0.81, 2.73)	1.20 (0.62, 2.33)	0.587
	Secondary education	26	72	1.27 (0.69, 2.38)	1.02 (0.52, 2.03)	0.945
	College and above	16	65	1.0	1.0	
Residence	Urban	46	167	0.45 (0.31, 0.69)	1.01 (0.59, 1.70)	0.982
	Rural	58	76	1.0	1.0	
History of preterm delivery	Yes	37	43	1.17 (1.13, 2.56)	0.81 (0.47 1.39)	0.447
	No	67	200	1.0	1.0	
History of abortion	Yes	41	37	2.38 (1.60, 3.520)	1.19 (0.69, 2.06)	0.516
	No	63	206			
The desire for a recent pregnancy	Unplanned	18	27	1.54 (0.93, 2.56)	1.70 (0.97, 3.02)	0.065
	Planned	86	216	1.0	1.0	
Chorioamnionitis	Yes	65	23	6.68 (4.47, 9.98)	3.89 (2.44, 6.18)	<0.001^*^
	No	39	220	1.0	1.0	
GDM	Yes	47	23	4.05 (2.77, 6.02)	2.01 (1.27, 3.17)	0.003^*^
	No	57	220	1.0	1.0	
Apgar scored in the fifth minute, less than 7	Yes	18	16	2.01 (1.26, 3.49)	1.87 (1.04, 3.36)	0.036^*^
	No	86	227	1.0	1.0	
RDS	Yes	55	50	2.67 (1.81, 3.93)	2.03 (1.14, 3.61)	<0.001^*^
	No	49	193	1.0	1.0	
Hypoglycemia	Yes	94	194	1.76 (0.91, 3.38)	1.28 (0.63, 2.63)	0.496
	No	10	47	1.0	1.0	
KMC	No	70	118	2.43 (1.60, 3.71)	1.86 (1.18, 2.94)	0.007^*^
	Yes	34	125	1.0	1.0	
Neonates entered an incubator	No	57	144	0.89 (0.60, 1.31)	1.37 (0.89, 2.10)	0.158
	Yes	47	129	1.0	1.0	
Gestational age at delivery in weeks	<32	28	21	4.86 (2.58, 9.11)	2.52 (1.27, 3.17)	0.008^*^
	32–34.6/7	61	127	2.36 (1.34, 4.15)	1.84 (1.01, 3.35)	0.045^*^
	35–36.6/7	15	95	1.0	1.0	

Abbreviations: GDM, gestational diabetes mellitus; KMC, kangaroo mother care; RDS, respiratory distress syndrome.

## 4 Discussion

This study assessed the survival status and predictors of mortality among preterm neonates admitted to the NICU in public hospitals in Central Ethiopia. The incidence rate of mortality was 39.88 (95% CI: 32.90–48.33) deaths per 1000 person‐day observations. Maternal chorioamnionitis, maternal gestational diabetes mellitus, low Apgar score at the fifth minute less than 7, having RDS, not receiving KMC, and gestational age less than 32 weeks during delivery were the predictors of mortality in preterm neonates.

The incidence rate of mortality observed in this study is in‐line with studies conducted in a tertiary care center in Sri Lanka (44.7%) [29], Addis Ababa public hospitals (36.4%), and Felege Hiwot Comprehensive Hospital (31%) in Ethiopia [24, 25]. In alignment with prior findings from Ethiopia, the highest incidence rate of mortality occurred within the first day of follow‐up [23]. This highlights the importance of giving more attention to immediate and intensive care during the initial postnatal period to improve survival outcomes.

In contrast, the incidence rate of mortality in this study is lower than those reported in the University Teaching Hospital of Butare, Rwanda (96%) [30]; the Ashanti region of Ghana (51.8%) [31]; and Mizan Tepi University Teaching Hospital in Ethiopia (62.15 deaths per 1000 person‐days observation) [23]. This variation may be explained by differences in patient profiles, as teaching hospitals often manage more severe or high‐risk cases compared to zonal or district‐level facilities. Conversely, our finding is higher than reports from those reported from high‐income countries, and data from Neonatal Research Network in the NEOCOSUR Centers (South American) show that survival rates for very low birth weight infants have significantly improved, with total neonatal mortality around 26.8% for infants born between 23 and 35 weeks of gestation [32], China [33] and Iran (9.1%) [34], Sierra Leone (20.7%) [35], Gondar (28.8%) [36], and Debre Markos Referral Hospital (27.11%) [37] in Ethiopia. The discrepancy may be attributed to differences in the level of neonatal care infrastructure. High‐income or better resourced settings, such as those in China and Iran, typically have access to advanced neonatal care technologies, surfactant therapy, and trained personnel, all of which are crucial for improving preterm survival. In contrast, the NICUs in our setting may face limitations in equipment, staffing, and clinical guidelines, contributing to the observed higher mortality rate [38].

This study's findings revealed that the hazard of death was two times more likely among preterm neonates with RDS than among neonates without RDS. A similar association of higher mortality among preterm neonates with RDS was reported in a previous study in Ethiopia [23, 27], Zimbabwe [39], and Brazil [40]. RDS in preterm neonates is related to surfactant deficiency, which predisposes them to alveolar collapse, hypoxia, and subsequent complications [41]. This risk may be exacerbated in our setting due to limited access to surfactant replacement therapy, mechanical ventilation, or continuous positive airway pressure devices. These findings underscore the potential value of implementing early surfactant administration protocols and expanding access to respiratory support technologies in low‐resource NICUs.

Consistent with previous study findings in Ethiopia [21, 24] and Uganda [42], preterm neonates who did not receive KMC had a higher hazard of death. KMC is a cost‐effective, evidence‐based intervention for preterm neonates, known to enhance thermoregulation by providing thermal care via continuous skin‐to‐skin contact, increasing the frequency of feeding intervals, reducing infections, and helping early recognition of illness [43]. Scaling up KMC coverage could serve as an effective strategy to improve survival among preterm infants in similar settings.

This study also demonstrated that the hazard ratio of death was 3.89 times more likely among neonates born to mothers with chorioamnionitis. The increased risk of neonatal mortality among infants born to mothers with chorioamnionitis has been reported in previous studies in the United States [44, 45]. This association may be due to increased neonatal exposure to systemic inflammation and infection.

Similarly, the hazard of death was two times more likely among neonates born to mothers with GDM. A previous study has shown that GDM moderately increased the risk of adverse perinatal outcomes [46]. These findings highlight the importance of antenatal screening and management of maternal infections and metabolic conditions.

Moreover, very/early preterm birth (<32 weeks’ gestation) had 2.5 times higher hazard of death than late preterm neonates. This could be due to a delay in lung maturation in early preterm neonates. Neonates born before 34 weeks of gestation miss the surfactant surge time. This delays lung maturation, resulting in increased respiratory distress, thereby increasing the hazard of death. A previous study in Ethiopia reported that when gestational age at birth increases by 1 week, the risk of mortality among preterm neonates is reduced by 28% [27]. This emphasizes the need for antenatal corticosteroid administration and timely delivery planning for women at risk of preterm birth.

We include all preterm neonates admitted to the NICU during the study period, and therefore, it is not prone to sampling errors. This study identified preventable neonatal and maternal predictors through a prospective follow‐up study. However, this study did not assess the appropriateness of treatments provided and delays in the management of those predictors.

## 5 Conclusions

The incidence rate of preterm neonatal mortality observed in this study was high. Significant predictors of mortality included maternal chorioamnionitis, maternal gestational diabetes mellitus, a fifth‐minute Apgar score below 7, the presence of RDS, lack of KMC, and gestational age less than 32 weeks at birth. To improve the survival of preterm neonates in public hospitals in central Ethiopia, targeted interventions should prioritize neonates with these risk factors. This includes enhanced monitoring and management of neonates with low Apgar scores, RDS, and those not receiving KMC, as well as improved antenatal care for mothers with chorioamnionitis or GDM. Additionally, regular audits of clinical practices and timely management of at‐risk neonates are essential to achieving the goal of ending preventable neonatal mortality.

### 5.1 Implications and Recommendations

This study identified key predictors for preterm mortality; it is vital to translate these findings into actionable interventions.

Recommendations include early identification and management of RDS with appropriate respiratory support, routine provision of KMC to all preterm infants, and strengthening antenatal care, with particular attention to the screening and management of maternal infections such as chorioamnionitis and metabolic disorders like GDM.

## Author Contributions

**Daniel Tsega:** conceptualization, investigation, supervision, resources, validation, writing – original draft, writing – review and editing, methodology. **Shegaw Geze Tenaw:** conceptualization, investigation, supervision, validation, writing – review and editing, writing – original draft. **Bogale Chekole:** conceptualization, data curation, formal analysis, investigation, writing – review and editing, writing – original draft, software. **Abdulaziz Assefa:** conceptualization, validation, data curation. **Mulugeta Animaw:** conceptualization, data curation, validation. **Aberash Beyene Derribow:** conceptualization, data curation, validation. **Mangistu Abera:** investigation, supervision, writing – review and editing. **Aynalem Belay:** investigation, supervision, writing – review and editing.

## Ethics Statement

This study was conducted following the Declaration of Helsinki guidelines for research. Ethical approval was obtained from the Research Ethical Review Committee of the College of Medicine and Health Sciences at Wolkite University. A formal support letter was submitted to each participating hospital, and permission was granted before data collection.

## Consent

Written informed consent was obtained from the mothers of the neonates before the study began. For neonates who died during their hospital stay, maternal agreement was sought at the time of admission or before the study began. To ensure participant confidentiality, data were collected anonymously.

## Conflicts of Interest

The authors declare no conflicts of interest.

## Data Availability

The data set used or analyzed during this study is available from the corresponding author upon reasonable request.
